# Coordinating Etching Inspired Synthesis of Fe(OH)_3_ Nanocages as Mimetic Peroxidase for Fluorescent and Colorimetric Self-Tuning Detection of Ochratoxin A

**DOI:** 10.3390/bios13060665

**Published:** 2023-06-19

**Authors:** Hongshuai Zhu, Bingfeng Wang, Yingju Liu

**Affiliations:** 1 College of Food Science and Technology, Henan Agricultural University, Zhengzhou 450003, China; 2 Key Laboratory for Biobased Materials and Energy of Ministry of Education, College of Materials and Energy, South China Agricultural University, Guangzhou 510642, China

**Keywords:** biomimetic nanozymes, coordinating etching, Fe(OH)_3_ nanocages, dual-mode fluorescence and colorimetric immunoassay, ochratoxin A detection

## Abstract

The development of multifunctional biomimetic nanozymes with high catalytic activity and sensitive response is rapidly advancing. The hollow nanostructures, including metal hydroxides, metal-organic frameworks, and metallic oxides, possess excellent loading capacity and a high surface area-to-mass ratio. This characteristic allows for the exposure of more active sites and reaction channels, resulting in enhanced catalytic activity of nanozymes. In this work, based on the coordinating etching principle, a facile template-assisted strategy for synthesizing Fe(OH)_3_ nanocages by using Cu_2_O nanocubes as the precursors was proposed. The unique three-dimensional structure of Fe(OH)_3_ nanocages endows it with excellent catalytic activity. Herein, in the light of Fe(OH)_3_-induced biomimetic nanozyme catalyzed reactions, a self-tuning dual-mode fluorescence and colorimetric immunoassay was successfully constructed for ochratoxin A (OTA) detection. For the colorimetric signal, 2,2′-azino-bis (3-ethylbenzothiazoline-6-sulfonic acid) diammonium salt (ABTS) can be oxidized by Fe(OH)_3_ nanocages to form a color response that can be preliminarily identified by the human eye. For the fluorescence signal, the fluorescence intensity of 4-chloro-1-naphthol (4-CN) can be quantitatively quenched by the valence transition of Ferric ion in Fe(OH)_3_ nanocages. Due to the significant self-calibration, the performance of the self-tuning strategy for OTA detection was substantially enhanced. Under the optimized conditions, the developed dual-mode platform accomplishes a wide range of 1 ng/L to 5 μg/L with a detection limit of 0.68 ng/L (S/N = 3). This work not only develops a facile strategy for the synthesis of highly active peroxidase-like nanozyme but also achieves promising sensing platform for OTA detection in actual samples.

## 1. Introduction

The colorimetric immunosensor has been supposed as a powerful technique to detect contaminants because of its inherent strength, such as simplicity of detection, low cost, and practicality [1,2,3]. Especially, this strategy can be recognized by human eyes without any sophisticated and expensive equipment [4]. For example, He et al. designed an ultra-sensitive visual method based on enzyme-induced gold nanoparticle aggregation to investigate the activity of alkaline phosphatase [5]. Wang et al. developed an online colorimetric acetone sensor by the specific reaction between hydroxylamine sulfate and acetone [6]. Mustafa proposed a highly specific enzyme-induced paper biosensor, which can predict fish freshness by monitoring the enzymatic conversion of hypoxanthine [7]. Moon et al. reported a colorimetric viral determination strategy in the light of the inter-spaced palindromic repeats (CRISPR)/Cas9 endonuclease dead system, which can successfully identify pH1N1/H275Y, pH1N1, and SARS-CoV-2 viruses through visualization [8]. Since the detection results can be judged visually without complex equipment, the colorimetric immunosensor has been used for on-site and portable detection [9,10,11]. Nevertheless, unsatisfactory sensitivity and accuracy severely hampers its further development and wide application in practical measurement [12,13]. Consequently, in order to enhance the detection selectivity and sensitivity, dual-mode immunosensor has been proposed recently [14,15,16,17]. In light of the chromogenic properties of oxidized *p*-phenylenediamine and its inner filter effect with organic silicon nanodots, Li et al. established a dual-mode fluorescent and colorimetric sensor to detect glucose in serum [18]. By employing giant Au vesicles as an intelligent probe, Guo et al. proposed a facile and efficient surface-enhanced Raman scattering and colorimetric dual-mode immunoassay for ultra-sensitive detection of *Vibrio parahaemolyticus* [19]. Notwithstanding, the dual-mode immunosensor can generate two different response signals and avoid false negative and positive results. Nevertheless, the two signals only respond linearly to the target concentration, and there is no correlation between the two detection results. Hence, in this work, according to the Pearson correlation analysis, the dynamic response relationship between two signals in a dual-mode immunosensor was investigated. Eventually, to a certain degree, the proposed strategy can eliminate the disturbance of environmental factors and enhance the sensitivity for trace analysis.

The performance of the immunosensor was basically decided by the catalytic ability of the enzyme. To date, the development of natural enzymes has been greatly restricted due to the high cost and poor environmental tolerance. Concurrently, with the rapid development of nano-materials in recent years, nanozymes have been supposed as a feasible substitute for natural enzyme because of its extraordinary merits including cost-effectiveness, easy preparation, mass-production, multiple functionalities, high stability, and durability [20,21,22]. Since the accidental discovery of Fe_3_O_4_ nanoparticles with peroxidase mimetic activity in 2007 [23], nanozymes have been reported for various applications. For instance, Chen et al. synthesized Co_3_O_4_@Co-Fe double-shelled nanocages by employing ZIF-67 as a starting template and then developed a colorimetric strategy for screening acetylcholinesterase activity by the high peroxidase mimetic activity of Co_3_O_4_@Co-Fe [24]. Mu et al. reported catalase mimics-like Co_3_O_4_ with different morphology for the determination of calcium ion [25]. Jin et al. prepared Si-doped CoO (Si-CoO) nanorods via hydrothermal strategy, which showed excellent peroxidase mimetic activity, to construct a colorimetric sensor for the detection of glutathione [26]. Commendably, in previous studies, there is an interesting finding that most of the nanozymes are concentrated in metal oxides [27], metals [28], metal organic framework (MOFs) [29], and carbons [30]. Few studies have investigated the application of metal hydroxides in the field of nanozyme catalysis [31]. Recently, 3D metal hydroxides have been demonstrated to exhibit promising catalytic activity due to their structural characteristics and tremendous catalytic active sites. Particularly, 3D hollow metal hydroxides with controllable morphology, size, and properties have received more attention.

**Scheme 1:**
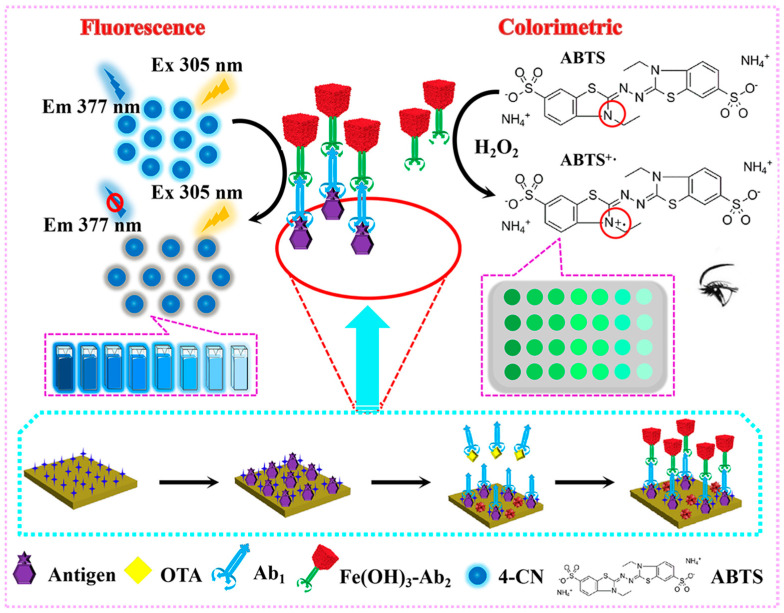
Schematic illustration of the self–tuning dual–mode immunosensor for OTA detection: fluorescence and colorimetry.

In this study, based on the coordinating etching principle, a facile template-assisted strategy was proposed for synthesizing Fe(OH)_3_ nanocages by using Cu_2_O nanocubes as the templates. Simultaneously, Fe(OH)_3_ nanocages were discovered to exhibit excellent properties of mimetic peroxidase. Fe(OH)_3_ nanocages were used to anchor the secondary antibody (Ab_2_) and then introduced into an immunosensor by an indirect immune competitive strategy. Thus, as in Scheme 1, a self-tuning dual-mode immunosensor was constructed by an Fe(OH)_3_-mediated Fenton-like reaction. Meanwhile, ochratoxin A (OTA), the most widely distributed ochratoxins, was used as the target analyte to demonstrate the performance of the developed immunosensor [32,33]. After the specific immune reaction, the immobilized Fe(OH)_3_@Ab_2_ in the 96-microplate can efficiently change the emission peak at 377 nm of 4-chloro-1-naphthol (4-CN) for the fluorescence signal, while, the non-immune bound Fe(OH)_3_@Ab_2_ can be used to catalyze the chromogenic substrate of 2,2′-azino-bis (3-ethylbenzothiazoline-6-sulfonic acid) diammonium salt (ABTS) with the assistance of H_2_O_2_ for the colorimetric signal. Consequently, the concentrations of OTA can be intuitively and conveniently identified. Ultimately, the fluorescence and colorimetric signals showed a direct negative correlation, which realized the self-calibration of the dual-mode sensor and greatly enhanced its accuracy and reliability.

## 2. Experimental Section

Materials and instruments are detailed in the Supporting Information.

### 2.1. Synthesis of Fe(OH)_3_ Nanocages

**Figure 1:**
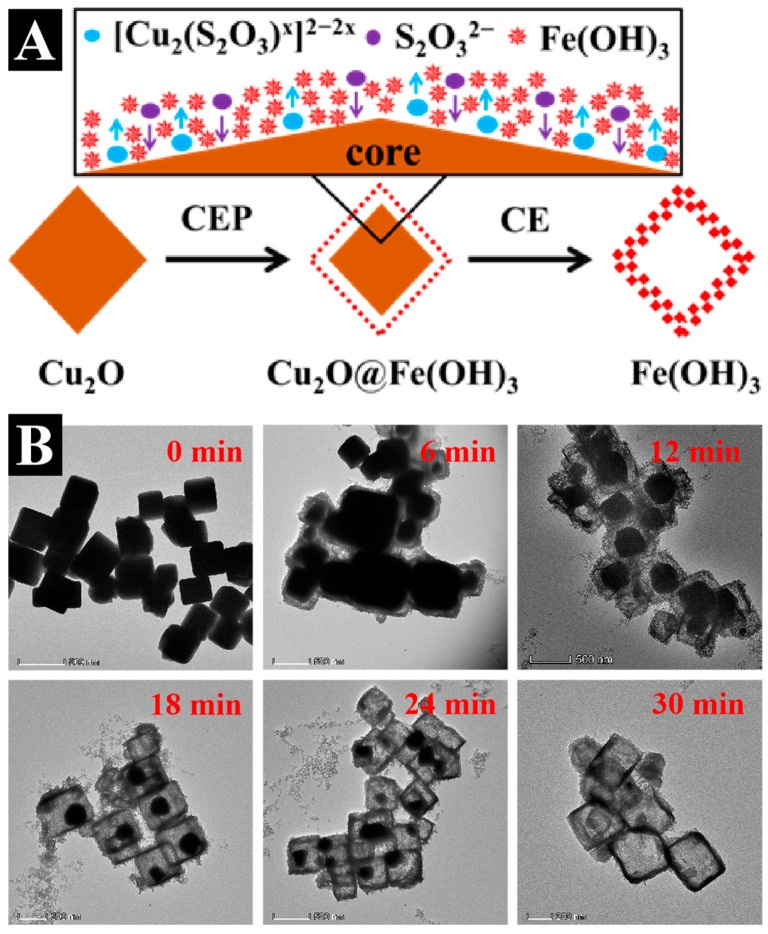
(**A**) Schematic diagram of Fe(OH)_3_ nanocages by the coordinating etching principle, CEP, coordinating etching and precipitating; CE, coordinating etching. (**B**) TEM images of the time-dependence of Fe(OH)_3_ nanocages for different times.

Fe(OH)_3_ nanocages were obtained according to Figure 1A. After Cu_2_O templates were prepared according to the earlier research [34], Fe(OH)_3_ nanocages were synthesized by removing the Cu_2_O cubic templates and precipitating Fe(OH)_3_ simultaneously. Briefly, 1.665 g polyvinylpyrrolidone was dissolved in 50 mL water, then FeCl_2_·4H_2_O (0.0105 g) and Cu_2_O templates (0.025 g) were dispersed by stirring for 10 min, followed by adding Na_2_S_2_O_3_ (5 mL, 0.6 M) at uniform speed and reacting for another 30 min. After washing by water and ethanol, the final product was dispersed in 10 mL methanol. All processes were carried out at room temperature and pH 10.

### 2.2. Peroxidase Kinetics of the Fe(OH)_3_ Nanozyme

The kinetic parameters of the Fe(OH)_3_ nanozyme were deduced by monitoring the adsorption of the mixture including the Fe(OH)_3_ nanozyme, H_2_O_2_, and ABTS at 415 nm with the time. First, in HAc-NaAc buffer (0.2 M, pH = 4), a fixed concentration of the Fe(OH)_3_ nanozyme (180 μM) and H_2_O_2_ (4.84 mM) was employed to study the kinetic parameters of ABTS by changing its concentration from 0.48 to 3.87 mM. Subsequently, the concentration of H_2_O_2_ was altered between 1.45 and 4.84 mM while unchanging that of ABTS (3.87 mM). Then, the kinetic constants were obtained by using the Lineweaver–Burk plot from the Michaelis–Menten equation, as below:

$$\frac{1}{V}=\left(\frac{1}{\left[S\right]}\right)\left(\frac{K_{m}}{V_{max}}\right)+\frac{1}{V_{max}}$$

where *V* is the initial velocity and [*S*] is the ABTS or H_2_O_2_ concentration. In addition, *K_m_* and *V_max_* are the Michaelis constant and the maximal reaction velocity, which can indicate the affinity between the Fe(OH)_3_ nanozyme and the substrate (ABTS or H_2_O_2_).

Next, the catalytic performance of the Fe(OH)_3_ nanozyme was further explored by theoretical calculation. The structure of small molecules involved in the decomposition of H_2_O_2_ were constructed by Gviewer 5.0, then geometry optimization was carried with the density functional theory (DFT) at M06-2X/CC-PVDZ basis set level [35,36]. There are two possible configurations of the d orbital electron in the ferric atom including high spin and low spin. It can be confirmed by calculation that the high spin configuration was tested to be more stable, so all the following calculations were set as high spin. According to the earlier report [37], the H_2_O_2_ decomposition mechanism was explored by calculating the energy change and transition state, so the difficulty degree of catalytic reaction was further demonstrated. As for the catalysis of the ferric ion on H_2_O_2_, the key step is the OH and O_2_^−^ radicals generated by the H_2_O_2_ with the valence transition of the ferrous ion in Fe(OH)_3_ nanocages. However, geometries of amorphous ferric oxide in the solvent are uncertain, so the numbers of water linked at the active position are uncertain, and several ferrous clusters may be connected together. Hence, a creative model was built, replacing the water molecule by dummy atoms. All calculations were finished with Gaussian 16 program. Data and figures including orbitals energy, orbital population, and ultraviolet frequencies were extracted by MultiWFN 3.7.

### 2.3. Preparation of Fe(OH)_3_@Ab_2_

Firstly, Fe(OH)_3_ nanocages were aminated by employing 7.2 mL of the above solution and 0.2 mL 3-aminopropyl trimethoxysilane (APTMS) to ethanol (35.6 mL), followed by stirring gently at 30 °C for 24 h, centrifuging and re-dispersing in Tris-HAc solution (10 mM). Next, the mixture (2 mL) was ultrasonicated at a low temperature for 20 min, and 4 μL Ab_2_ (1 mg/mL) was injected and stirred gently at 4 °C for 24 h. Finally, the mixture was centrifuged at a low temperature and re-dispersed in PBS (2 mL) for further use. Therefore, based on the covalent binding between amino groups of Fe(OH)_3_ nanozyme and carboxyl groups of the second antibody (Ab_2_), the Fe(OH)_3_@Ab_2_ immune complex was synthesized for target recognition and signal output.

### 2.4. The Construction of the Self-Tuning Immunoassay

Briefly, 50 μL dopamine (1 mg/mL) was injected into the micro-plate and incubated at 37 °C for 30 min. After drying, the OTA antigen (30 μL, 10 μg/mL) was injected and held at 37 °C for 2 h. Consequently, in order to seal up the rest of the unspecific sites, 30 μL blocking mixture was added. Subsequently, the solution (30 μL) containing various concentrations of OTA and a fixed amount of antibody (Ab_1_, 5 μg/mL) was added to each well, then kept at 37 °C for 60 min (each well was washed by PBST and dried with N_2_ after each step). Next, the Fe(OH)_3_@Ab_2_ solution (30 μL) was injected and kept at 37 °C for 60 min, and the unconjugated solution was collected. Finally, 20 μL PBST was injected into each well to wash the microplate, which was collected and mixed with the above unconjugated solution.

Afterwards, for the fluorescence signal, after the sandwich immunoreaction, 150 μL of 4-CN (0.4 mM) was transferred into the above microplate and reacted for 4 min. The fluorescent intensity of the mixture was monitored by a fluorescence spectrometer. Additionally, for the colorimetric signal, 30 μL unconjugated mixed solution, 200 μL of pH 4.5 HAc-NaAc buffer (0.2 M), 30 μL ABTS (30 mM), and 50 μL H_2_O_2_ (35 mM) were sequentially added in a new 96-well plate. After reacting at 25 °C for 3 min, the solution color was changed into green and photographed by an iPhone 12, while the absorption intensity was monitored by a UV–vis spectrophotometer.

## 3. Results and Discussion

### 3.1. Characterization of Fe(OH)_3_ Nanocages

According to the coordinating etching principle, a facile strategy was developed to synthesize Fe(OH)_3_ nanocages by carefully controlling the precipitation rate of Fe(OH)_3_ and the coordinating etching rate towards the sacrificial templates of Cu_2_O (Figure 1A) [38]. The reaction between Fe^2+^ and Cu_2_O follows Pearson’s hard and soft acid–base principle, where soft acids prefer to form stable complexes with soft bases, while hard Lewis acids prefer hard bases. Leveraging the soft acid properties of Cu^+^ within the Cu_2_O templates, the coordinating etchant S_2_O_3_^2−^ was chosen. Initially, S_2_O_3_^2−^ formed soluble and stable [Cu_2_(S_2_O_3_)_x_]^2−2x^ complexes on the Cu_2_O surface. Simultaneously, a significant amount of OH^−^ was generated from the interaction of S_2_O_3_^2−^ with water during the etching process. Consequently, OH^−^ and Fe^2+^ accumulated around Cu_2_O, gradually forming a Fe(OH)_2_ shell. Under the experimental conditions, the formed Fe(OH)_2_ was oxidized to the more stable Fe(OH)_3_. This was evident from the maroon color observed in Figure S3. The size of the Cu_2_O nanocubes decreases over time while retaining their cubic morphology, ultimately leading to the fabrication of nanocage structures.

**Figure 2:**
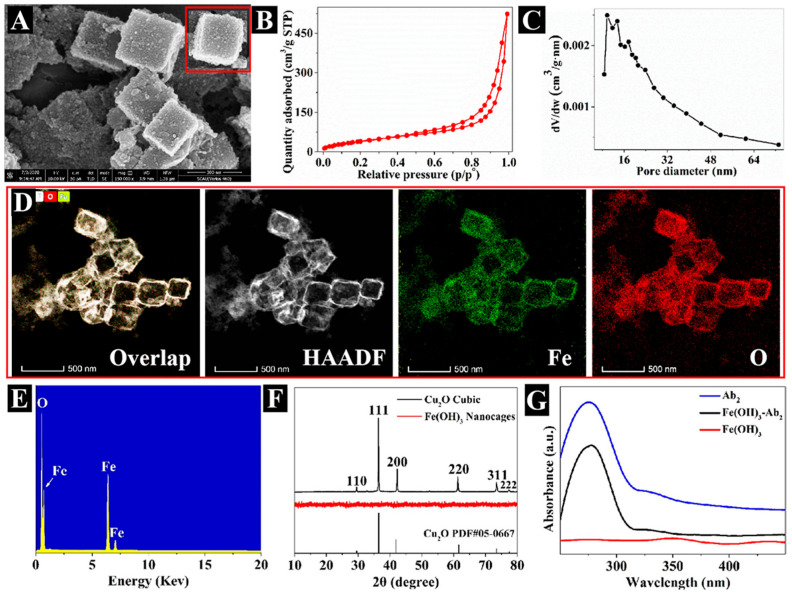
(**A**) SEM, (**B**) N_2_ absorption–desorption isotherm, (**C**) pore size distribution, (**D**) elemental mappings, (**E**) EDS, and (**F**) XRD of Fe(OH)_3_ nanocages. (**G**) UV–vis spectra of Fe(OH)_3_@Ab_2_.

To further verify these conclusions, transmission electron microscopy (TEM) was employed to demonstrate the whole etching process. As shown in Figure 1B, after 6 min of the etching reaction, the generated Fe(OH)_3_ nanosheets adhered to Cu_2_O nanocubes to form a stable film-like structure. The etching speed of Cu_2_O nanocubes far exceeded the formation speed of Fe(OH)_3_ nanosheets, so a cavity structure was clearly formed between Cu_2_O and Fe(OH)_3_ when the reaction time was prolonged to 12 min. Subsequently, with the extension of the etching time, it can be clearly observed that the Cu_2_O cores gradually decreased until it was completely etched, and the nanocages were successfully synthesized. In addition, from the formation process of nanocage structure, it can be found that the [Cu_2_(S_2_O_3_)^x^]^2−2x^ and S_2_O_3_^2−^ can easily flow in through the Fe(OH)_3_ shells by inter-particle interstitials, which may be due to the well-distributed pore size of formed Fe(OH)_3_ (12.27 nm, Figure 2C). In addition, the uniform porous structure of Fe(OH)_3_ nanocages can increase the friction of its contact surface, which prolongs the contact time between Fe(OH)_3_ and the substrate. The interesting nature of Fe(OH)_3_ nanocages can enormously accelerate the passage of reactive substrates and products into or out of the channels, and simultaneously provide more accessible active sites. Additionally, as shown in Figure 2B of the N_2_ absorption–desorption curve, the Brunauer–Emmett–Teller (BET) surface area exhibited obvious increase from 2 m^2^/g for Cu_2_O (Figure S1) to 134 m^2^/g for Fe(OH)_3_ nanocages. Consequently, Fe(OH)_3_ nanocages can provide more catalytic active sites and increase the rate of substrate diffusion [26]. Thus, the nanozyme activity and catalytic efficiency were substantially enhanced.

In order to further investigate the surface and internal structures of the generated Fe(OH)_3_ nanocages, scanning electron microscopy (SEM) and TEM photographs of Fe(OH)_3_ nanocages and Cu_2_O precursors were analyzed. As depicted in Figure S2A, it can be observed that monodisperse Cu_2_O exhibited smooth surface, uniform particle size, and stable tetragonal structure. When the etching process completed, the prepared Fe(OH)_3_ nanocages formed a sheet-like saddle-backing surface (Figure 2A), but it still kept a complete tetragonal spatial structure. Such unique appearance may also be the main reason for the significant increase in the surface area-to-mass ratio of Fe(OH)_3_. In addition, it can provide a very convenient condition for the attachment of bio-molecules and the reaction of chromogenic substrates.

Furthermore, compared with Cu_2_O (Figure S2D), the elemental mappings of Fe(OH)_3_ (Figure 2D) revealed that O and Fe were well distributed on the Fe(OH)_3_ surface. Additionally, from the energy dispersive spectrometry (EDS) of Fe(OH)_3_ (Figure 2E) and Cu_2_O (Figure S2C), it can be demonstrated that only O and Fe elements were distributed on Fe(OH)_3_ nanocages, which reflected that Cu_2_O nanocubes were completely etched. From the X-ray diffraction (XRD) spectrum in Figure 2F, the (220), (200), (110), (111), and (311) crystal planes of a cubic Cu_2_O can be found at 61.3°, 42.3°, 29.6°, 36.4°, and 73.5° (JCPDS 05-0667). However, when the Fe(OH)_3_ nanocages were formed, it became amorphous material [26].

**Figure 3:**
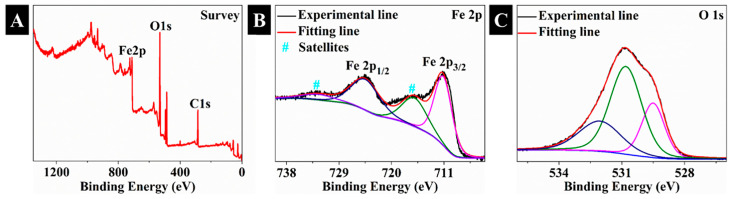
XPS survey (**A**), and the deconvolution of Fe 2p (**B**) and O 1s (**C**) of Fe(OH)_3_ nanocages.

Finally, the chemical composition of Fe(OH)_3_ nanocages was further investigated by X-ray photo-electron spectroscopy (XPS). From Figure 3A, it can be found that two binding energy bands at 284.4 eV and 529.9 eV attributed to C 1s and O 1s, respectively. Simultaneously, two adjacent peaks centered at 724.5–725.8 eV and 709.8–712.4 eV belonged to Fe 2p3 and Fe 2p1, respectively [39]. Then, the oxidation states of Fe and O in Fe(OH)_3_ nanocages were further studied. As shown in Figure 3B, based on the earlier work [38], the major peaks at 711.1 and 724.6 eV in the Fe 2p corresponded to Fe 2p_3/2_ and 2p_1/2_, which may be Fe^3+^ in Fe(OH)_3_. Additionally, the peaks at 716.4 and 731.8 eV corresponded to the satellite peaks. As depicted in Figure 3C of O 1s, the peak at 529.5 eV also reflects the existence of the high valence state of Fe^3+^. The major peak at 530.8 eV indicated the existence of hydroxyl ions, while the weakly adsorbed oxygen species can be found at the peak at 532.2 eV [38]. Thus, the results revealed that the final synthetic product should be Fe(OH)_3_ nanocages.

### 3.2. Catalytic Activity of Fe(OH)_3_ Nanocages

**Figure 4:**
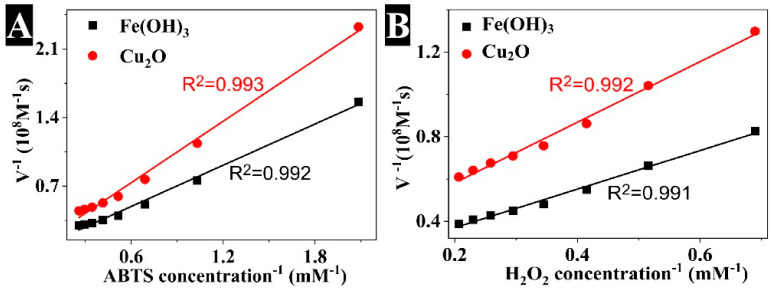
Lineweaver−Burk plots of Fe(OH)_3_ nanocages and Cu_2_O nanocubes by tuning the substrate of ABTS at a fixed 4.84 mM H_2_O_2_ (**A**), and tuning the H_2_O_2_ concentration at a fixed 3.87 mM ABTS (**B**), respectively.

The peroxidase-like behaviors of the Fe(OH)_3_ nanozyme were studied by varying the concentrations of ABTS at a certain concentration of H_2_O_2_ or varying concentrations of H_2_O_2_ at a certain volume of ABTS. As seen from Figure 4A,B, the kinetic parameters of Fe(OH)_3_ nanozyme and Cu_2_O were calculated by the Lineweaver–Burk plots, indicating that Fe(OH)_3_ presented a lower *K_m_* (0.7 mM) than that of Cu_2_O (0.77 mM) and horseradish peroxidase (HRP) (0.81 mM) [40]. Therefore, Fe(OH)_3_ exhibited a stronger affinity for ABTS and H_2_O_2_.

Compared with Cu_2_O, the unique nanocage-shaped structure of Fe(OH)_3_ nanocages can provide a higher BET surface area, leading to more catalytic active sites for H_2_O_2_, which was confirmed by the BET data (Figure 2B,C). Thus, the catalytic decomposition process of H_2_O_2_ can be more effective and quicker. More importantly, Liu et al. demonstrated that the reduction reaction of Fe^3+^/Fe^2+^ can effectively decompose H_2_O_2_ to generate OH radicals and O_2_^−^ radicals [12]. Both OH radicals and O_2_^−^ radicals can rapidly oxidize the chromogenic substrate ABTS to form a vivid color, which can be easily recognized by the human eye. Subsequently, Fe(OH)_3_ nanocages would have strong activity to convert H_2_O_2_ into OH radicals and H_2_O and thus exhibit excellent catalytic activity, which, in turn, results in a lower *K_m_*.

**Figure 5:**
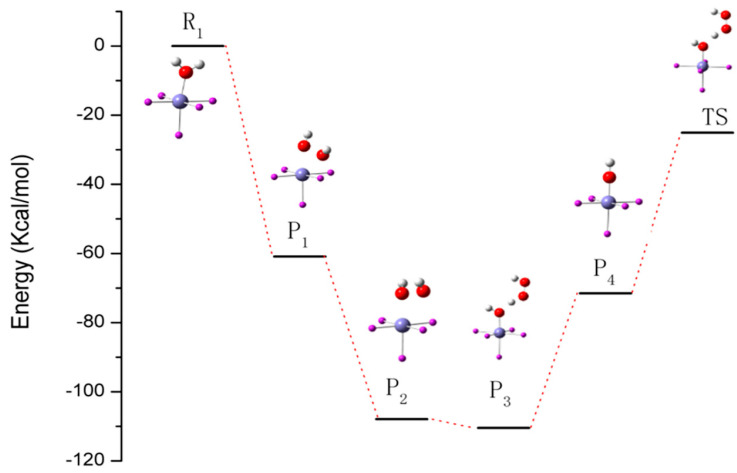
Fe(OH)_3_ catalyst reaction mechanism and energy change (insets are the structure of relevant molecules (ions); the center is iron ion, purple is ghost atom, red is oxygen atom, and white is hydrogen atom).

The excellent peroxidase mimetic activity of the Fe(OH)_3_ nanozyme was further verified by density functional theory (DFT). As depicted in Figure 5, it is defined that Fe (II) bounded with one water molecule as the zero potential start point (R_1_). When water molecule is replaced by hydrogen peroxide (P_1_), two both oxygen atoms are close to ferrous atom (0.208 nm). When Fe (II) turned to Fe (III) (P_2_), the bond length of Fe-O is 0.205 nm. Furthermore, another oxygen deviates from ferrous atom. The bond length of O-O in H_2_O_2_ is elongated to 0.143 nm and dihedral angle increases from 91.6° to 99.7°. Therefore, it can be found that when iron is oxidized from II to III, H_2_O_2_ begins to decompose into ⋅OH radicals and H_2_O. It can be demonstrated that Fe(OH)_3_ nanozyme is easier to catalyze H_2_O_2_ to produce OH radical from the marked decline of the reaction system energy. Hydroxyl bounded to ferrous atom (P_3_) reacts with H_2_O_2_, producing water and HO_2_ radical (P_4_). Finally, ⋅OH radicals can react with another H_2_O_2_ and detaches from P_4_, while the transition state (TS) was found by searching the reaction. Ultimately, the site is available for water and R_1_ is recycled. Hydrogen atom is captured by OH radicals, so the O-H bond length is extended from 0.099 nm to 0.112 nm and the O-O bond length is shortened. By calculating energies of every stage, H_2_O_2_ decomposition with the Fe(OH)_3_ nanozyme easily takes place, indicating that the theoretical results are consisted with the Lineweaver–Burk plot.

### 3.3. Characterization of the Fe(OH)_3_@Ab_2_ Bioconjugate

The aminated Fe(OH)_3_ nanocages were further studied in Figure S3 by a Fourier Infrared Spectrometer (FTIR). The obvious bands at 584 and 3346 cm^−1^ were from Fe-O and O-H stretching vibrations in Fe(OH)_3_, respectively. The other two characteristic bands at 952 and 1046 cm^−1^ were assigned to the N-H deformation vibration and C-NH_2_ stretching vibration, respectively. Additionally, the deformation and stretching vibrations of C-H were distributed at 1410 and 2936 cm^−1^, respectively. Consequently, the UV–vis was further employed to investigate the formation of Fe(OH)_3_@Ab_2_. As depicted from Figure 2G, the characteristic absorption of protein only can be found in Fe(OH)_3_@Ab_2_ [30], demonstrating that the Fe(OH)_3_ nanocages were successfully modified by Ab_2_.

### 3.4. The Feasibility of the Fe(OH)_3_ Nanocages-Induced Immunoassay

The fabrication of the Fe(OH)_3_ nanocages-induced immunoassay was illustrated in Scheme 1. Briefly, the coated antigen and different concentrations of OTA could competitively bind to the certain amount of Ab_1_ in the 96-well plates. Subsequently, the Ab_1_ can specifically bind with Fe(OH)_3_@Ab_2_, which can rapidly quench the fluorescence of 4-CN. Significantly, the fluorescence quenching effect may be attributed to the oxidation of 4-CN caused by the valence transition of the ferric ion in Fe(OH)_3_ nanocages [41,42]. Subsequently, the uncombined Fe(OH)_3_@Ab_2_ was collected for the colorimetric detection, where the Fe(OH)_3_ can catalyze the oxidation of ABTS to generate a vivid color change and UV–vis absorption intensity variation. Based on the mechanism of the designed self-tuning sensor, a trade-off was developed between the colorimetric and fluorescence signal, as one falls, the other is rising. It bears a striking resemblance to the balance of the two opposing principles in nature from the Book of Changes. Additionally, the signal acquisition of the fluorescence and colorimetric immunosensor was separated, and the detection results did not interfer with each other. Thus, the accuracy and reliability of the self-tuning immunosensor can be significantly enhanced.

Subsequently, in order to further demonstrate the feasibility of the designed platform, the controlled experiment of the developed self-tuning fluorescence and colorimetric immunosensor was performed. As indicated from Figure S4 in the Supporting Information, as for the fluorescence signal, the fluorescence peak of 4-CN at 377 nm has no obvious change as the time prolonged (Figure S4A, curves a and b). However, when Fe(OH)_3_ nanocages were added to the reaction system, the fluorescence emission peak of 4-CN decreased significantly (Figure S4A, curve c), demonstrating that the Fe(OH)_3_-induced reaction can efficiently reduce the fluorescence intensity of 4-CN. Thus, the fluorescence signaling mechanism can be used for the sensitive and rapid detection of the target. For the colorimetric detection, when ABTS and H_2_O_2_ were in solution, the color of the reaction system has no significant change (inset in Figure S4B, curves a and b). As shown in Figure S4B, only when the Fe(OH)_3_ nanocages were added, the typical absorption spectrum of substrate ABTS can be found at 415 nm, exhibiting that Fe(OH)_3_ nanocages can effectively catalyze the chromogenic substrate to form a vivid color change (inset in Figure S4B, curve c). This colorimetric platform can be highlighted by its naked eye readout without complicated instruments. To summarize, the designed self-tuning immunosensor is reasonable and practicable enough to achieve the desired detection performance; furthermore, it is even simple in manipulation and fabrication compared to traditional detection tools.

### 3.5. Self-Tuning Colorimetric and Fluorescence Immunoassay of OTA

To achieve an optimum analytical performance, the relevant factors were optimized, such as the concentration of H_2_O_2_, reaction time, the excitation wavelength, and quenching time of 4-CN. Experimentally, the colorimetric detection performance was determined by investigating the absorbance change at characteristic 415 nm of the oxidized ABTS (oxABTS) product. The influence of H_2_O_2_ concentration was shown in Figure S5A. As the H_2_O_2_ concentration increased from 5 to 35 mM, the absorption peak at 415 nm of the system also enhanced remarkably. When the H_2_O_2_ concentration was higher than 35 mM, the absorbance intensity of oxABTS did not increase remarkably. As seen from Figure S5B, the absorbance intensity of oxABTS also increased rapidly with the extension of reaction time, but after 3 min, it entered a relative steady phase. Therefore, the 35 mM of H_2_O_2_ concentration and 3 min reaction time were selected for the subsequent experiments. Additionally, as in Figure S5C, it can be seen that the change of 4-CN fluorescence intensity reached an equilibrium at 4 min. Then, with the further extension of time, the reduction of 4-CN fluorescence intensity was not obvious. Hence, the optimal quenching time was set as 4 min for the following experiments.

**Figure 6:**
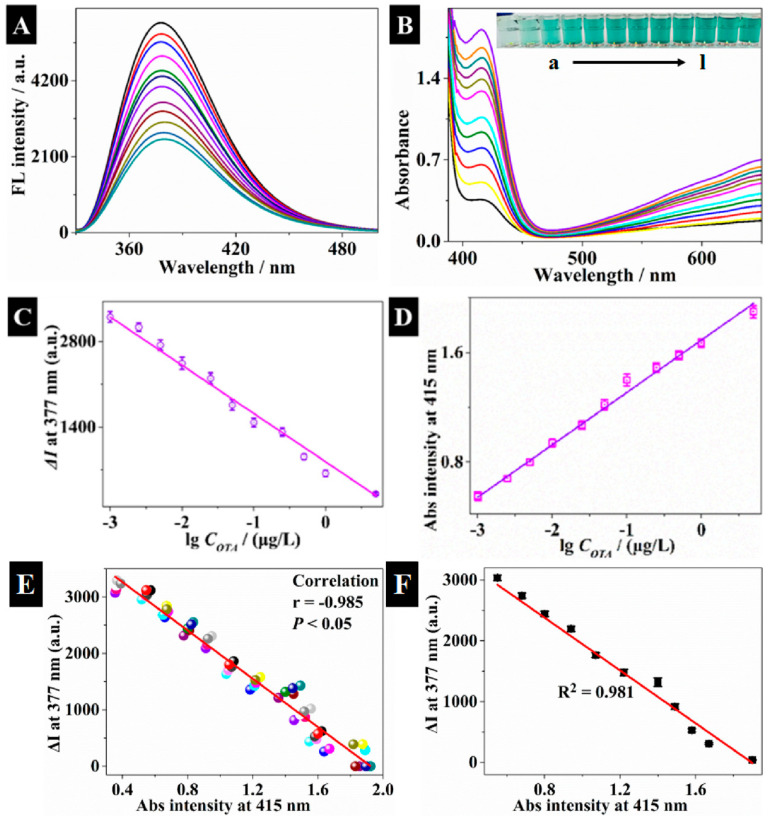
Fluorescence spectra (**A**) and UV–vis spectra (**B**) for different concentrations of OTA, (a) 0, (b) 0.001, (c) 0.0025, (d) 0.005, (e) 0.01, (f) 0.025, (g) 0.05, (h) 0.1, (i) 0.25, (j) 0.5, (k) 1, and (l) 5 μg/L (from bottom to top in A and B correspond to a–l, respectively). Inset in B was the photographs of the colorimetric method at different concentrations of OTA. (**C**,**D**) Calibration curve based on the fluorescence spectra (**A**) and UV–vis spectra (**B**), respectively. (**E**,**F**) Pearson correlation analysis of the self–tuning immunosensor (**E**) and corresponding calibration curve (**F**).

Under the optimized conditions, the self-tuning immunosensor was constructed for OTA detection in light of colorimetric and fluorescence signals. As displayed in Figure 6A,B, when the OTA concentration increased, the UV–vis absorption intensity of the colorimetric signal increased while the quenching degree of 4-CN fluorescence intensity decreased. Concurrently, with the change in the OTA concentration, the immunosensor can generate a vivid color change that could be preliminarily identified by the naked eye (inset in Figure 6B). Simultaneously, the UV–vis absorption intensity and fluorescence intensity changes exhibited a good linear relation in the range of OTA concentrations between 1 ng/L and 5 μg/L with a detection limit (0.68 ng/L, S/N = 3) (Figure 6C,D), where the regression equations were Abs_415_ = 1.7 + 0.4[lg*C*_OTA_ (μg/L)] (R^2^ = 0.995, colorimetric signal) and Δ*I*_377nm_ (a.u.) = 725.7 − 853.6[lg*C*_OTA_ (μg/L)] (R^2^ = 0.986, fluorescence signal), respectively. Additionally, compared with other peroxidase or current OTA detection methods (Table S1), the designed self-tuning strategy based on the Fe(OH)_3_ nanozyme had equivalent or better performance in the linear range or detection limit. Therefore, the significant self-calibration capability of this self-tuning detection platform can provide a more sensitive and accurate performance, particularly in OTA detection of actual samples.

To further demonstrate the substantial capability of the proposed self-tuning dual-mode immunosensor, the Pearson’s correlation coefficient test was conducted to verify the correlation between fluorescence (Δ*I*, the quenching level of fluorescence intensity at 377 nm) and colorimetric (Abs_415_, the UV–vis absorbance intensity at 415 nm) signals (*p* < 0.05 was regarded as significant difference). The results were summarized in Figure 6E, where a negative correlation was disclosed between Δ*I* and Abs_415_ with a Pearson’s correlation coefficient of −0.985 (*p* < 0.05). Concurrently, as reflected from Figure 6F, the Abs_415_ of the colorimetric detection showed a good linear relationship with Δ*I* changes of the fluorescence detection in the range of OTA concentration between 1 ng/L and 5 μg/L, while the linear fitting equation was Δ*I* = −4116.4 + 2170.5[Abs_415nm_ (a.u.)] (R^2^ = 0.981). The statistical analysis further demonstrated that the two signals of the proposed self-tuning immunosensor can be mutually corrected, which substantially enhanced the accuracy and reliability of the detection results.

**Figure 7:**
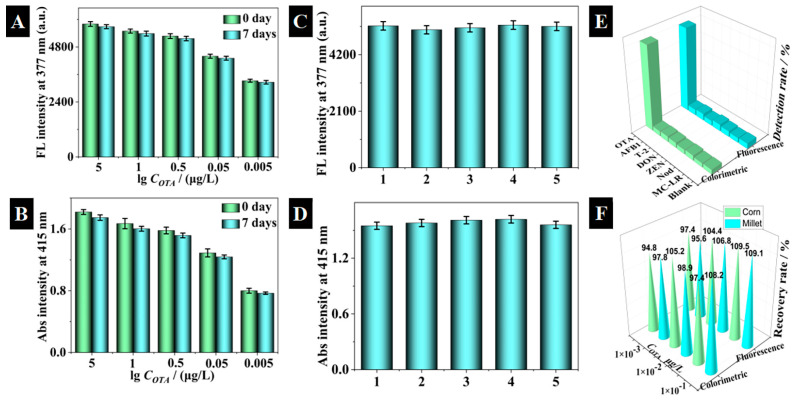
The stability (**A**,**B**) and reproducibility (**C**,**D**) of the self–tuning immunosensor, (**E**) selectivity of the self–tuning dual–modal immunosensor, and (**F**) the recoveries of the OTA detection in corn and millet samples.

As for the actual application, an immunosensor should be not only sensitive but also stable and reproducible. Hence, to investigate the stability of the proposed sensor, as depicted in Figure 7A,B, the long-term stability of the strategy was evaluated with the same immunosensor using five OTA concentrations. After a week of storage, the fabricated sensor can retain sufficient stability for the detection of OTA. Subsequently, the reproducibility of the strategy was also demonstrated by employing five independent immunosensors with the same concentration of OTA (Figure 7C,D). As for colorimetric or fluorescence detection, there is basically no difference between five sensors, which demonstrated that the designed self-tuning immunosensor exhibited desirable stability and reproducibility for OTA detection.

Additionally, the interference investigation was conducted using microcystin-LR (MC-LR), nodularin (Nod), aflatoxin B_1_ (AFB_1_), zearalenone (ZEN), deoxynivalenol (DON), and trichothecenes 2 (T-2). As seen in Figure 7E, the interferents had no significant influence on the self-tuning strategy for the OTA detection (both the colorimetric and fluorescence immunoassay), indicating the presented self-tuning immunosensor had excellent specificity to the target detection. Subsequently, in order to demonstrate the analytical reliability of the immunosensor and further investigate its potential practical application. Thus, the standard addition experiments were conducted by spiking the actual corn and millet samples at different concentrations of OTA standard solution (0.001, 0.01, and 0.1 μg/L). As depicted in Figure 7F, the recoveries of the self-tuning immunosensor were in a preferable range of 94.8–108.2% (colorimetric sensor) and 95.6–109.5% (fluorescence sensor), respectively. Hence, the developed self-tuning strategy has a promising performance for OTA detection in application.

## 4. Conclusions

In this work, a coordinating etching facile strategy was proposed to synthesize Fe(OH)_3_ nanocages by employing Cu_2_O nanocubes as the sacrificial templates. It was demonstrated that Fe(OH)_3_ nanocages resulted in the enhanced catalytic activity and followed the Lineweaver–Burk plot and Michaelis–Menten equation for the enzyme-catalyzed ABTS/H_2_O_2_ reaction. The enhanced peroxidase-like activity was mainly due to the large surface of the Fe(OH)_3_ nanocages and the decrease in the reaction system energy, which was confirmed by the BET surface area and density functional theory. Subsequently, based on the high peroxidase-like activity of the Fe(OH)_3_ nanocages, a self-tuning colorimetric and fluorescence strategy was designed for OTA detection. Due to the see-saw effect of signal modes, the two signals can be verified by each other directly to enhance the detection accuracy. Hence, OTA can be detected at a lower concentration of 0.68 ng/L, and the immunosensor showed excellent recovery rates of 94.8–109.5% in corn and millet samples with acceptable reproducibility and stability. In summary, this work not only investigates the feasibility of coordination etching for the synthesis of nanozymes with excellent peroxidase catalytic activity, but also broadens the detection platform for complex mycotoxin samples.

## Data Availability

The data presented in this study are available on request from the corresponding author.

## Supplementary Materials

The following supporting information can be downloaded at: https://www.mdpi.com/article/10.3390/bios13060665/s1, Figure S1: N_2_ absorption–desorption isotherm of Cu_2_O nanocubes; Figure S2: (A) SEM image, (B) TEM image, (C) EDS, (D) elemental mappings of Cu_2_O nanocubes; Figure S3: FTIR spectrum of Fe(OH)_3_-NH_2_ (inset illustration is russet color of the Fe(OH)_3_ nanocages); Figure S4: Feasibility control experiments of the designed self-tuning fluorescence (A, a and b were 4-CN with the reaction times of 0 and 4 min, respectively; c was the mixture of 4-CN with Fe(OH)_3_ with the time at 4 min) and colorimetric immunosensor (B, a: HAc-NaAc buffer (pH = 4.5)+ ABTS; b: HAc-NaAc buffer (pH = 4.5) + ABTS + H_2_O_2_; c: HAc-NaAc buffer (pH = 4.5) + ABTS + H_2_O_2_ + Fe(OH)_3_, respectively); Figure S5: Effect of the concentration of H_2_O_2_ (A) and the reaction time (B) on the colorimetric part. Effect of the quenching time of 4-CN (C) on the fluorescence part; Table S1: Comparison with other peroxidase or current OTA detection methods [43,44,45,46,47,48,49,50,51,52,53,54].
