# Supplementary material for: Coordinating Etching Inspired Synthesis of Fe(OH)3 Nanocages as Mimetic Peroxidase for Fluorescent and Colorimetric Self-Tuning Detection of Ochratoxin A
Source: Biosensors (Basel). 2023 Jun 19;13(6):665. doi: 10.3390/bios13060665 (PMC10296677; doi:10.3390/bios13060665)
Supplement: Supplementary file 1 [file biosensors-13-00665-s001.zip › biosensors-2395903-supplementary.pdf]

biosensors MDPI

*Supplementary Material*

# Coordinating Etching Inspired Synthesis of $Fe(OH)_3$ Nanocages as Mimetic Peroxidase for Fluorescent and Colorimetric Self-Tuning Detection of Ochratoxin A

**Hongshuai Zhu [1,2,†], Bingfeng Wang [2,†] and Yingju Liu [2,*]**

[1] College of Food Science and Technology, Henan Agricultural University, Zhengzhou 450003, China
[2] Key Laboratory for Biobased Materials and Energy of Ministry of Education, College of Materials and Energy, South China Agricultural University, Guangzhou 510642, China
* Correspondence: yingjuliu@scau.edu.cn; Tel.: +86-20-85280319; Fax: +86-20-85285026
† These authors contributed equally to this work.

## 1. Materials and Instruments

Cupric chloride dihydrate ($CuCl_2·2H_2O$), ascorbic acid (AA), sodium hydroxide (NaOH), hydrochloric acid, sodium acetate, sodium thiosulfate, and $H_2O_2$ (30%) were re-ceived from Aladdin biological technology Co. Ltd (Shanghai, China). 4-CN was received from Beijing InnoChem Science & Technology Co. Ltd (Beijing, China) and dissolved in ethanol at a certain concentration. Polyvinylpyrrolidone (PVP, MW: 40, 000) was bought from Beijing Solarbio Science & Technology Co. Ltd (Beijing, China). ABTS was purchased from Sigma Aldrich Chemical Co. Ltd (Shanghai, China). Tween and dopamine hydro-chloride were purchased from Acros Organics (Shanghai, China). Iron (II) chloride tetra-hydrate ($FeCl_2·4H_2O$) and 3-aminopropyl trimethoxysilane (APTMS) were received from Shanghai Macklin Biochemical Co. Ltd (Shanghai, China). The antigen of ochratoxin A (OTA, 5 mg/mL) and the antibody of OTA ($Ab_1$, 1 mg/mL) were from College of Food Sciences, South China Agricultural University (Guangzhou, China). The secondary goat anti-rabbit antibody ($Ab_2$, 1 mg/mL) was bought from Santa Cruz Biotechnology (Shang-hai, China). The washing buffer was 0.01 M PBS (pH 7.4) with 0.5% tween-20 (PBST pH 7.4, 0.01 M).

The morphologies of nanomaterials were demonstrated by scanning electron micros-copy (SEM, Talos F200S, Thermo Fisher, Waltham, America), transmission electron mi-croscopy (TEM, Talos L120C, Thermo Fisher, Waltham, America), automatic rapid spe-cific surface area and porosity analyzer (ASAP-2460, Micromeritics, Atlanta, America), while the structure and element analysis of nanomaterials were characterized by X-ray diffraction (XRD, D/max-IIIA, Rigaku Corporation, Tokyo, Japan), X-ray photo-electron spectroscopy (XPS, K-Alpha+, Thermo Fisher, Waltham, America), Fourier Infrared Spec-trometer (FTIR, Nicolet IS 10, Thermo Fisher, Waltham, America) and Energy Dispersive Spectrometer (EDS, SS550&SEDX-550, Shimadzu, Kyoto, Japan). The absorption spectrum was recorded by an UV-visible spectrophotometer (Evolution 220, Thermo Fisher, Wal-tham, America). The fluorescence spectra were performed by a fluorescence spectrometer (F-7000, Hitachi, Tokyo, Japan).

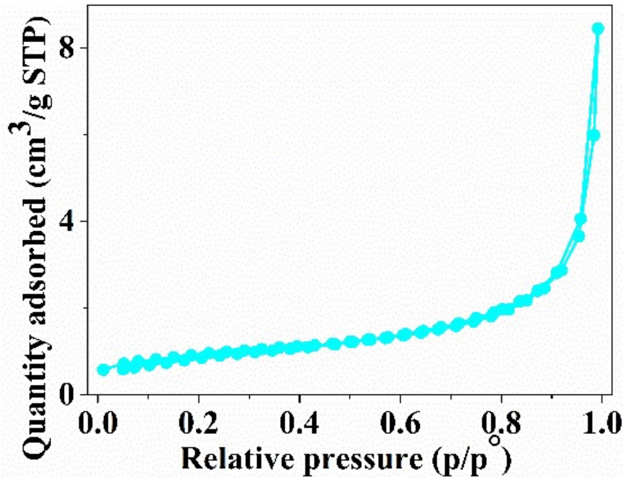


**Figure S1.** $N_2$ absorption-desorption isotherm of $Cu_2O$ nanocubes.

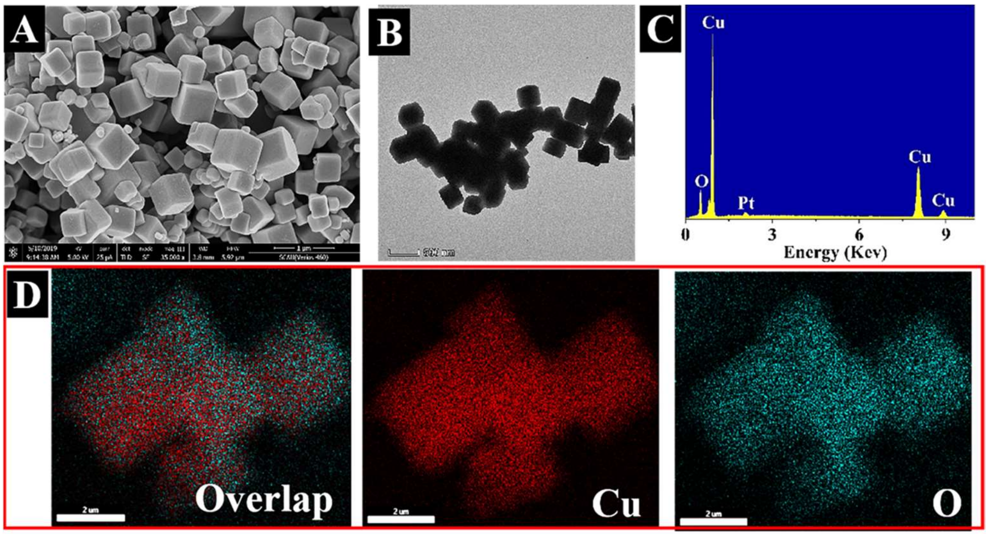


**Figure S2.** (A) SEM image, (B) TEM image, (C) EDS, (D) Elemental mappings of $Cu_2O$ nanocubes.

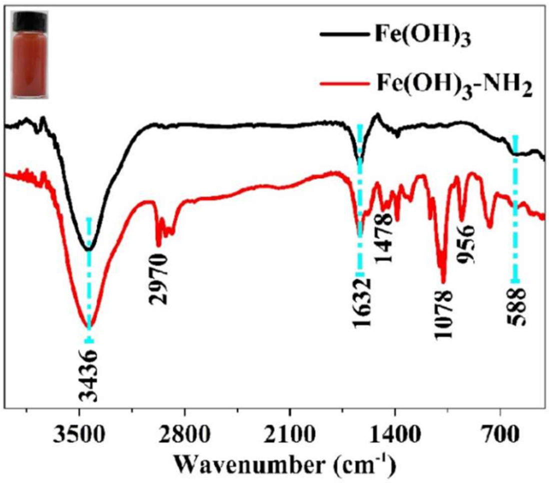


**Figure S3.** FTIR spectrum of $Fe(OH)_3$-$NH_2$ (inset illustration is russet color of the $Fe(OH)_3$ nanocages).

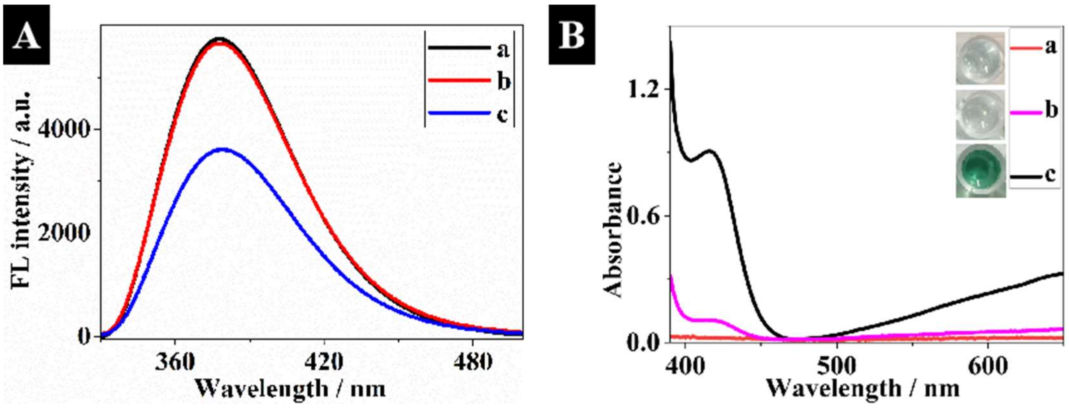


**Figure S4.** Feasibility control experiments of the designed self-tuning fluorescence (A, a and b were 4-CN with the reaction times of 0 and 4 min, respectively; c was the mixture of 4-CN with $Fe(OH)_3$ as the time at 4 min) and colorimetric immunosensor (B, a: HAc-NaAc buffer (pH=4.5)+ ABTS; b: HAc-NaAc buffer (pH=4.5) + ABTS + $H_2O_2$; c: HAc-NaAc buffer (pH=4.5) + ABTS + $H_2O_2$ + $Fe(OH)_3$, respectively).

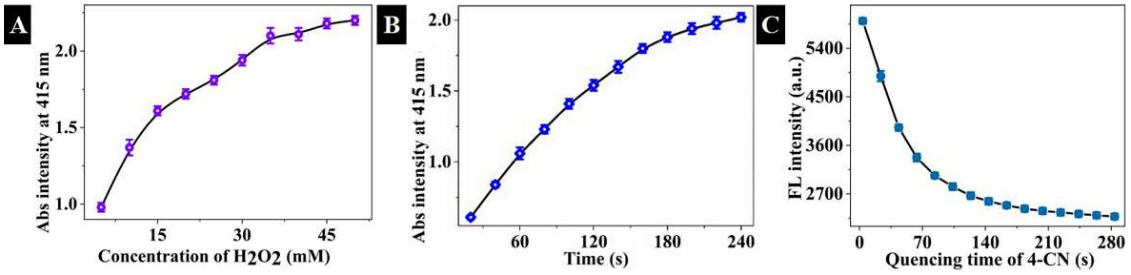


**Figure S5.** Effect of the concentration of $H_2O_2$ (A) and the reaction time (B) on the colorimetric part. Effect of the quenching time of 4-CN (C) on the fluorescence part.

**Table S1.** Comparison with other peroxidase or current OTA detection methods.

| Detection method | LOD | Linear range | Reference |
|---|---|---|---|
| Aptasensor | 33.3 ng/mL | 0.1–100 μg/mL | [1] |
| Fluorescence assay | 1.86 ng/mL | 5–100 ng/mL | [2] |
| Lateral flow immunoassay | 0.094 μg/L | 0.098–12.5μg/L | [3] |
| Aptasensor | 3.96 ng/mL | 4.04–80.76 ng/mL | [4] |
| Fluorescence sensor | 3.513 ng/mL | 4.038–20.19 ng/mL | [5] |
| Electrochemical sensor | 5.65 ng/mL | 20.19–403.81 ng/mL | [6] |
| Electrochemical immunosensor | 2.57 ng/mL | 5–160 ng/mL | [7] |
| Ratiometric aptasensor | 380 ng/L | 5 μg/L–700 μg/L | [8] |
| Electrochemical/visual aptasensor | 25.2 fg/mL | 0.1–200 ng/mL | [9] |
| Electrochemical sensor | 0.03 ng/mL | 0.1–100 ng/mL | [10] |
| Electrochemical sensor | 0.5 ng/L | 1 ng/L–1 μg/L | [11] |
| Ratiometric aptasensor | 3.4 ng/mL | 0.01–50 ng/mL | [12] |
| Colorimetric immunoassay | 0.75 ng/L | 1 ng/L–5 μg/L | This work |
| Fluorescence immunoassay | 0.68 ng/L | | |

## References


1. Chen, J.; Wei, Q.; Yang, L.; Li, J.; Lu, T.; Liu, Z.; Zhong, G.; Weng, X.; Xu, X. Multimodal ochratoxin A-aptasensor using 3'-FAM-enhanced exonuclease I tool and magnetic microbead carrier. *Anal. Chem*. **2022**, *94*, 10921–10929.
2. Wu, S.; Liu, L.; Duan, N.; Wang, W.; Yu, Q.; Wang, Z. A test strip for ochratoxin A based on the use of aptamer-modified fluorescence upconversion nanoparticles. *Mirochim. Acta* **2018**, *185*, 497.
3. Hao, L.; Chen, J.; Chen, X.; Ma, T.; Cai, X.; Duan, H.; Leng, Y.; Huang, X.; Xiong, Y. A novel magneto-gold nanohybrid-enhanced lateral flow immunoassay for ultrasensitive and rapid detection of ochratoxin A in grape juice. *Food Chem*. **2021**, *336*, 127710.
4. Lv, L.; Li, D.; Cui, C.; Zhao, Y.; Guo, Z. Nuclease-aided target recycling signal amplification strategy for ochratoxin A monitoring. *Biosens. Bioelectron*. **2017**, *87*, 136–141.

5. Wang, C.; Tan, R.; Chen, D. Fluorescence method for quickly detecting ochratoxin A in flour and beer using nitrogen doped carbon dots and silver nanoparticles. *Talanta* **2018**, *182*, 363–370.
6. Pacheco, J.; Castro, M.; Machado S.; Barroso M. F.; Nouws H. P. A.; Delerue-Matos, C. Molecularly imprinted electrochemical sensor for ochratoxin A detection in food samples. *Sensor. Actuat. B: Chem.* **2015**, *215*, 107–112.
7. Hu, S.; Ouyang, W.; Guo, L.; Lin, Z.; Jiang, X.; Qiu, B.; Chen, G. Facile synthesis of $Fe_3O_4$/g-$C_3N_4$/HKUST-1 composites as a novel biosensor platform for ochratoxin A. *Biosens. Bioelectron*. **2017**, *92*, 718–723.
8. Zeng, H.L.; Ma, L.; Xia, X.H. G-quadruplex specific dye-based ratiometric FRET aptasensor for robust and ultrafast detection of toxin. *Dyes Pigm.* **2019**, *164*, 35–42.
9. Zhang, X.; Zhi, H.; Zhu, M.; Wang, F. Electrochemical/visual dual-readout aptasensor for ochratoxin A detection integrated into a miniaturized paper-based analytical device. *Biosens. Bioelectron*. **2021**, *180*, 113146.
10. Li, X.; Chen, X.; Huang, H. Development of a novel label-free impedimetric electrochemical sensor based on hydrogel/chitosan for the detection of ochratoxin A. *Talanta* **2021**, *226*, 122183.
11. Wang, X.; Gong, M.; Jin, X.; Jiang, M.; Xu, J. A novel electrochemical sensor for ochratoxin A based on the hairpin aptamer and double report DNA via multiple signalamplification strategy. *Sensor Actuat. B-Chem*. **2019**, *281*, 595–601.
12. Guo, J.; Pan, L.; Wang, M.; Chen, L.; Zhao, X. Exogenous interference and autofluorescence-free ratiometric aptasensor for detection of OTA based on dual-colored persistent luminescence nanoparticles. *Food Chem.* **2023**, *413*, 135611.

**Disclaimer/Publisher's Note:** The statements, opinions and data contained in all publications are solely those of the individual author(s) and contributor(s) and not of MDPI and/or the editor(s). MDPI and/or the editor(s) disclaim responsibility for any injury to people or property resulting from any ideas, methods, instructions or products referred to in the content.
